# Rates of Smoking Cessation at 6 and 12 Months after a Clinical Tobacco Smoking Cessation Intervention in Head and Neck Cancer Patients in Northern Ontario, Canada

**DOI:** 10.3390/curroncol29030130

**Published:** 2022-03-02

**Authors:** Stacey A. Santi, Michael S. C. Conlon, Margaret L. Meigs, Stacey M. Davidson, Kyle Mispel-Beyer, Deborah P. Saunders

**Affiliations:** 1 Health Sciences North Research Institute (HSNRI), Sudbury, ON P3E 5J1, Canada; ssanti@hsnri.ca (S.A.S.); mconlon@hsnri.ca (M.S.C.C.); pemeigs@hsnsudbury.ca (M.L.M.); kmispelbeyer@hsnsudbury.ca (K.M.-B.); 2 School of Rural and Northern Health, Laurentian University, Sudbury, ON P3E 2C6, Canada; 3 Northern Ontario School of Medicine (NOSM), Sudbury, ON P3E 2C6, Canada; stdavidson@hsnsudbury.ca; 4 Northeast Cancer Centre (NECC), Health Sciences North, Sudbury, ON P3E 5J1, Canada

**Keywords:** smoking cessation, head and neck cancer, nicotine dependence, tobacco, intensive clinical tobacco intervention

## Abstract

Smoking during cancer treatment is associated with reduced treatment response and cancer recurrence in patients with tobacco-related cancers. The purpose of this study was to examine smoking characteristics in head and neck cancer patients (*n* = 503) with a history of smoking and examine the impact of an intensive clinical tobacco intervention to patients who were currently smoking. All participants completed an interviewer-administered questionnaire at study enrollment which examined smoking behaviours, motivations to quit, and strategies used to cessate smoking. Follow-up assessments were completed at 6- and 12-months which monitored whether patients had quit smoking, remained cessated, or continued to smoke since study recruitment. For those who were currently smoking (*n* = 186, 37.0%), an intensive clinical tobacco intervention that utilized the 3A’s—Ask, Advise, Arrange—and the Opt-Out approach was offered to assist with smoking cessation at their new patient visit and followed-up weekly during their head and neck radiation therapy for 7 weeks. At 6 months, 23.7% (*n* = 41) of those who were smoking successfully quit; 51.2% quit ‘cold turkey’ (defined as using no smoking cessation assistance, aids or pharmacotherapy to quit), while 34.9% used pharmacotherapy (varenicline (Champix)) to quit. On average, it took those who were smoking 1–5 attempts to quit, but once they quit they remained cessated for the duration of the study. Although the head and neck cancer patients in this study reported high levels of nicotine dependence, many were able to successfully cessate.

## 1. Introduction

Continued smoking in patients who have been diagnosed and receive treatment for head and neck cancer is associated with decreased survival, higher rates of primary tumours [1], less successful treatment outcomes, and complications during cancer treatment [2,3]. Although the benefits of successful smoking cessation particularly during cancer treatment are known, high rates of smoking within this cancer population still persist, as well as high rates of relapse after a smoking cessation attempt [4]. Thus, a targeted smoking cessation intervention within this clinical population remains vitally important.

The purpose of the present study was to characterize the smoking behaviours and examine the impact of an intensive clinical tobacco intervention in a population of head and neck cancer patients who were being treated at the Northeast Cancer Centre (NECC) in Northeastern Ontario, ON, Canada. This geographical region is characterized by a large rural population, and has some of the highest reported smoking rates, as well as some of the highest lung cancer rates (another tobacco-related cancer) relative to the entire province of Ontario [5]. All current patients who were smoking and those with a smoking history who had cessated prior (former or ex-smokers) completed questionnaires at study enrollment [5], and then completed follow-up assessments at 6 and 12 months to monitor their current smoking status (still smoking or quit smoking for those who were smoking, or still cessated or had resumed smoking for those who had cessated). All current patients who smoke were offered an intensive clinical tobacco intervention on their new patient visit and followed-up weekly during their head and neck radiation therapy for 7 weeks. The program utilized the 3A’s—Ask, Advise, Arrange—and the Opt-Out [6] approach to assist with smoking cessation, and patients were also provided with motivational interviewing, and access to pharmacotherapy, nicotine replacement therapy, or combination therapy. Previously, we have published the smoking characteristics of the baseline cohort of this prospective study [5]. The findings reported herein outline the study outcomes, cessation rates, smoking characteristics, as well as relapse rates, at 6 and 12 months after the implementation of a clinical tobacco smoking cessation intervention program.

## 2. Materials and Methods

### 2.1. Study Population

The study cohort (*n* = 503) was comprised of patients diagnosed with head and neck cancer who attended the Northeast Cancer Centre Dental Oncology Clinic (Sudbury, ON, Canada) from December 2010 through to June 2020 (the initial study cohort and baseline data have been previously described [5], where the study data recruitment up to and including April 2018 was outlined). A consort chart of the original study recruitment is available here (Figure 1 in Conlon et al., 2020 [5]). Since that time, an additional *n* = 10 head and neck cancer patients were eligible for final inclusion into the study; *n* = 3 current smokers and *n* = 7 ex-smokers. An updated consort chart for the 6- and 12-month timepoints is presented in Supplementary Materials (Figure S1). Briefly, all patients were screened for their smoking history and ever-smokers were identified out of the study population by asking the question “have you ever smoked at least 100 cigarettes in your entire life?” The inclusion criteria for the study were: eligible ever-smokers who were at least 18 years of age or older, able to read/write/understand English, and capable of providing informed consent. Exclusion criteria for the study included patients with human immunodeficiency virus or hepatitis C as pre-existing medical conditions. All participants granted access to their medical records for the associated demographic and health variables (marital status, physical comorbidities, psychiatric comorbidities, alcohol consumption, alcohol dependency, and drug use) required for this study which were abstracted from medical chart reviews and physician notes. With respect to these variables, comorbidities were coded as ‘yes/no’ or ‘data not available’ if patients were receiving treatment or had a medical history of diabetes, hypertension, heart problems/heart failure, lung/COPD/asthma, stroke, other cancers, depression, anxiety, alcohol consumption, alcohol dependency, or drug use. All study participants consented to future contact from the investigators, if required. Buccal, saliva, or blood samples were also donated for SNP (single nucleotide polypmorphism) and genetic analyses. In total, this cohort included *n* = 503 head and neck cancer patients who were ever-smokers. Of these, *n* = 186 (37.0%) were currently smoking, and *n* = 317 (63.0%) were persons with a history of smoking (had cessated prior). In addition, all patients who were currently smoking were offered an Intensive Tobacco Intervention (described below) to assist with smoking cessation. Approval for this study was granted by the Health Sciences North Research Ethics Board (REB) (Study #755, approval date 7 February 2022).

### 2.2. Tobacco Use Questionnaires

All study participants (*n* = 503) completed 3 interviewer-administered questionnaires that assessed their past and current smoking characteristics and tobacco use. Items on the questionnaires were adapted from the Canadian Tobacco Use Monitoring Survey [7], and the National Enhanced Cancer Surveillance Study [8]. The Fagerström Test for Nicotine Dependence [9] was used to measure nicotine dependence, heaviness of smoking index [10], and time to first cigarette [11] as outlined in the initial study cohort and baseline data publication [5]. The first questionnaire was administered in-person by an interviewer and completed at the initial study enrollment. The study cohort at enrollment and the results of the smoking characteristics obtained on the baseline questionnaire have been previously published [5]. The two remaining questionnaires were administered at the 6- and 12-month follow-up timepoints via telephone assessment by the original interviewer (Supplementary Data, Table S1).

At the 6- and 12-month timepoints, all ever-smokers (both those currently smoking and those who cessated prior (ex-smokers) were questioned about their current smoking status. Abstinence was measured at the study follow-ups (6 and 12 months) by asking all participants if they were either smoking or had cessated since their enrollment into the study (baseline study timepoint). For those who were smoking at the baseline timepoint we asked if (a) they were still smoking since their initial questionnaire was completed, or (b) if they had since cessated smoking. Those who were smoking at the baseline timepoint and who had quit were asked about the strategies they used to quit, the number of attempts that they needed in order to quit successfully, the date they had quit, if they were experiencing any cravings (yes/no) to smoke and if so, the intensity of their urges to smoke (none, mild, moderate, or extreme). If they were still smoking, they were asked about the amount they were smoking, if they were interested in quitting smoking, and questioned about the strategies that they believed would assist them in quitting in the future. Similarly, those who had cessated prior (ex-smokers) were asked if they (a) still remained cessated since the beginning of the study, or (b) if they had since resumed smoking. If they had resumed smoking again, they were asked about the factors that lead them to re-start smoking and if they were interested in quitting again. We also asked those who had remained cessated about their cravings (yes/no) and urges (none, mild, moderate, or extreme) to have a cigarette on a regular basis in the questionnaire (the questionnaire used at the 6- and 12-month follow-up is available in Supplementary Table S1).

### 2.3. Intensive Clinical Tobacco Intervention

All newly diagnosed head and neck cancer patients who were currently smoking and attending the Northeast Cancer Centre Dental Oncology Clinic, were screened for tobacco use. An Intensive Clinical Tobacco Intervention was carried out by trained staff certified by the Centre for Addiction and Mental Health/Training Enhancement in Applied Counselling (CAMH/TEACH), and the Canadian Network of Respiratory Care (CNRC). The Intensive Clinical Tobacco Intervention was offered to all patients who were currently smoking regardless of the patient’s study enrollment status (patients who were not eligible for this study were still offered the assistance from this program). This program counselled patients on smoking cessation (3A’s—Ask, Advise, Arrange) [12,13,14] and utilized The Opt-Out approach [6,15] as well as nicotine replacement therapy (NRT), pharmacotherapy, or combination therapy treatment options to aid those currently smoking in their smoking cessation efforts [5]. Patients who chose to participate in the program were followed weekly while they were receiving radiation treatment for their head and neck cancer (duration of active treatment) and the Clinical Tobacco Intervention was combined with these follow-up visits for 8 weeks (including the new patient visit). The weekly follow-ups included assessment of oral toxicities, pain management, and the clinical tobacco intervention included follow-up of patients’ smoking status, counselling (3A’s), motivational interviewing and assessed adherence to pharmacotherapy, NRT, combination therapy, or cold turkey. Our study follow-ups were scheduled for 6 months and 1 year later after the date of initial study enrollment [5] to assess smoking cessation status.

### 2.4. Statistical Analysis

Descriptive statistics and frequency counts were used to analyze the sociodemographic data, smoking characteristics, cancer stage, and ICD diagnoses for the study. Logistic regression analyses were used to calculate the odds ratios (ORs), adjusted odds ratios (adj ORs), and 95% confidence intervals (CIs) for the smoking characteristics (comparing those patients who were currently smoking and those who had a history of smoking (ex-smokers)). Age, sex, marital status, psychiatric comorbidities, alcohol dependency, alcohol consumption and drug use were used as the covariates for the adjusted analysis. Frequency counts and descriptive statistics were utilized for the data analysis of current smoking behaviours obtained from the 6- and 12-month questionnaires, where comparisons were made between participants who were still smoking and those who were cessated, or where the data were examined as differences between the 6- and 12-month follow-ups. All data were analyzed using SPSS v.24.

## 3. Results

### 3.1. Study Cohort and Sociodemographics

A total of 503 head and neck cancer patients who were ever-smokers and reported to the Clinical Tobacco Intervention Program for treatment between December 2010 and June 2020 were enrolled in this study. We have previously published the smoking characteristics of the baseline timepoint which outlined the study recruitment that occurred from December 2010 through to April 2018 [5]. Since that time interval, recruitment into the study continued until June 2020 and the final number of eligible patients with completed assessments enrolled in this study increased from 493 to 503 (an additional *n* = 10; where *n* = 3 were currently smoking, and *n* = 7 were cessated prior). Out of this total, 186 (37.0%) reported that they were currently smoking while the remaining 317 (63.0%) identified as having a prior smoking history (had cessated prior), similar to what was reported for the baseline cohort (37.1%) [5]. As previously reported [5], the majority of our study cohort was male (77.3%, *n* = 389), 67 years of age (median), and most reported that they were married or in a long-term relationship (63.8%, *n* = 321) (Table 1). In terms of the clinical correlates, most of the head and neck cancers were classified as cancers of the oral cavity (ICD diagnosis C01–C06, 37.0%, *n* = 186), and diagnosed at stage T2 (30.6%, *n* = 154). Hypertension was noted as the most frequent comorbidity in our head and neck cancer patients (27.7%, *n* = 196), and most (91.8%, *n* = 462) reported no psychiatric comorbidities such as anxiety and depression. More than half of the study population consumed alcohol (59.6%, *n* = 300), with those currently smoking reporting higher levels of alcohol consumption than ex-smokers (65.1% compared to 56.5% for those who had been cessated) (Table 1). While only 12.3% (*n* = 62) of the total sample reported alcohol dependency, a higher proportion occurred in those who were currently smoking (22.6%, *n* = 42; Table 1).

### 3.2. Smoking Characteristics in the Study Population

Previously, we reported that the head and neck cancer patients in this study had long smoking durations, higher smoking pack years, and higher numbers of cigarettes smoked per day, particularly for those who were currently smoking [5]. As anticipated, neither the additional recruitment of patients since 2018, nor the additional variables used in the adjusted odds ratio analyses (marital status, psychological comorbidities, alcohol consumption, alcohol dependency and drug use) substantially changed the findings that were initially reported for our baseline cohort (Table 2). Of those who were smoking in our sample, 52.7% (*n* = 98) had been smoking for 44 years or more, and compared to ex-smokers, showed higher smoking pack years (adj OR = 6.16; 95% CI 3.34–11.36) and higher numbers of cigarettes smoked per day (adj OR = 3.70; 95% CI 1.79–7.65). At baseline, 29.2% (*n* = 147) of the total sample showed ‘high’ dependence scores on the Fagerstrom Test for Nicotine Dependence, and those currently smoking were more likely to report higher nicotine dependence levels (adj OR ranging from 3.73–5.45) and a higher heaviness of smoking index (adj OR ranging from 2.57–3.52) than those who had cessated, as previously reported [5] (Table 2). The majority of the sample, regardless of if they were smoking or had cessated prior, reported a time to first cigarette of 30 min or less (70.8%, *n* = 356).

### 3.3. Cessation Rate after an Intensive Clinical Tobacco Intervention

**Figure 1:**
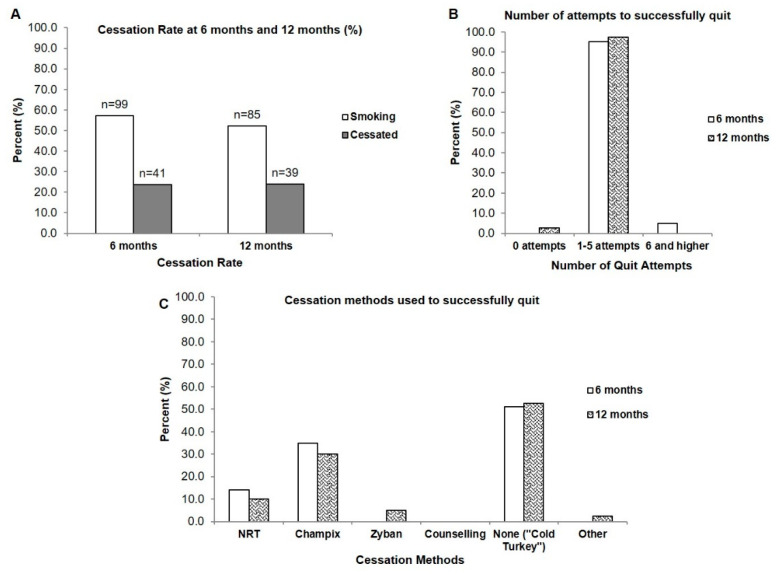
Cessation rate, number of quit attempts, and cessation methods of those currently smoking who cessated after presentation of a clinical tobacco intervention. (**A**) Cessation rate at 6- and 12-months (**B**) Number of quit attempts required by those who were smoking for successful cessation (*n* = 41 at 6 months; *n* = 39 at 12 months) (**C**) Cessation methods used to quit (*n* = 41 at 6 months; *n* = 39 at 12 months).

At the 6-month follow-up, 41 patients who were smoking had successfully quit after undergoing the intensive clinical tobacco intervention, with a cessation rate of 23.7% (*n* = 173, as 13 patients were deceased prior to the 6-month intake) (Figure 1A). At 12 months, the cessation rate was 23.9% (*n* = 163, excluding patients who were deceased after the 6-month follow-up) (Supplementary Materials Figure S1). On average, it took those currently smoking between 1–5 attempts to successfully quit and this was consistent at both the 6-month and 12-month follow-up; with the exception of a small percentage that required 6 attempts and higher to successfully cessate (4.9%) (Figure 1B). At 6 months, the majority (51.2%) of those who were smoking quit using no additional cessation methods or products (i.e., ‘cold turkey’ which was defined as using no smoking cessation aids or pharmacotherapy to assist with quitting smoking). The remainder used varenicline (Champix) (34.9%) and nicotine replacement therapy (14.0%) (Figure 1C). Interestingly, the smokers who quit self-reported that they used no counselling methods to quit even though they would have received counselling at the cancer clinic from the Intensive Clinical Tobacco Intervention (Figure 1C).

### 3.4. Cravings and Urges to Smoke on a Regular Basis in Smokers Who Quit and in Those Who Had Been Cessated

**Figure 2:**
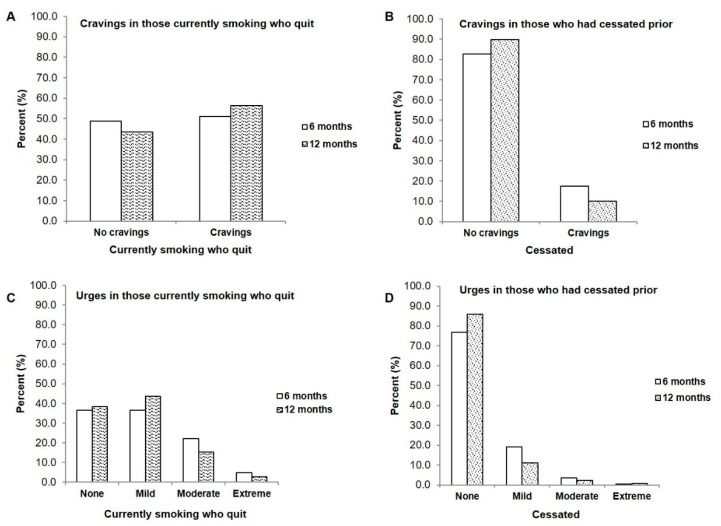
Comparison of cravings and urges between patients who were smoking and who quit after enrollment in a clinical tobacco intervention and those who were already cessated at the start of the study (**A**) Cravings in those smoking who quit, at 6 months (*n* = 41) and 12 months (*n* = 39) (**B**) Cravings in those who were cessated at 6 months (*n* = 281) and 12 months (*n* = 276) (**C**) Urges to smoke in patients who were smoking and quit at 6- and 12-months compared to (**D**) Urges to smoke in those who had already cessated.

When current smokers who had quit were questioned about their cravings to smoke on a regular basis, the results were equally divided with 48.8% reporting no cravings to smoke and 51.2% reporting regular cravings at the 6-month timepoint (Figure 2A). The large majority of those who had cessated prior reported no cravings to smoke on a regular basis (82.6% at 6 months and 89.9% at 12 months (Figure 2B)). A similar trend was observed in urges to smoke, with those who recently quit experiencing various levels of urges primarily ranging from ‘no urges’ (36.6% at 6-months) to ‘moderate urges’ (22.0%, 6-months, Figure 2C). Those who had been cessated prior (ex-smokers) primarily reported feeling no urges to smoke at all on a regular basis (76.9%, 6-months, Figure 2D).

### 3.5. Participants Who Continued to Smoke

**Figure 3:**
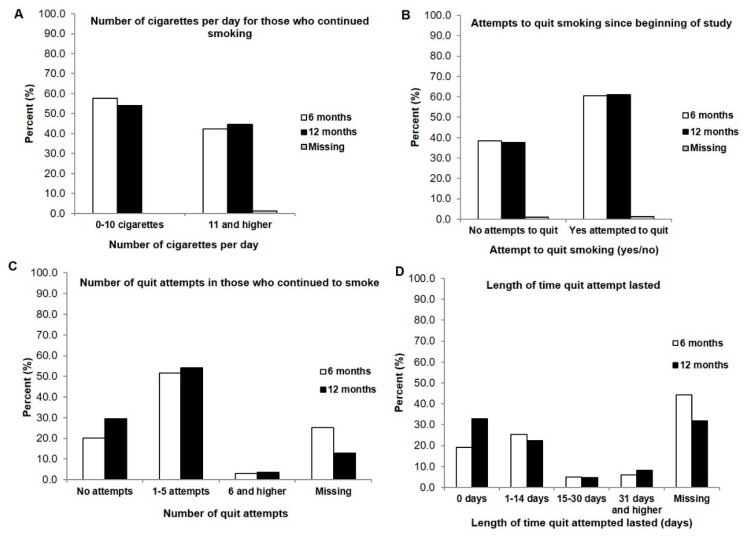
Participants who continued to smoke after presentation of a clinical tobacco intervention at 6 months (*n* = 99) and 12 months (*n* = 85) (**A**) Number of cigarettes smoked per day at 6 months and 12 months (**B**) Attempted to quit at 6 months and 12 months (yes/no) (**C**) Number of quit attempts made by those who smoke at 6- and 12-months (**D**) Length of time quit attempt lasted before beginning to smoke again at 6 and 12 months.

When we examined the smoking characteristics in those patients who continued to smoke after being presented with the clinical tobacco intervention, some surprising findings emerged. Despite still continuing to smoke (Figure 3A), over 60% had tried to quit smoking at both the 6- and 12-month follow-ups (Figure 3B), and most (approximately 50%) made between 1 and 5 quit attempts (Figure 3C). Each of these quit attempts lasted from 0 days (19.2% to 32.9% at 6- and 12-months) to 14 days (25.3% and 22.4%) (Figure 3D). However, when we questioned those who were still actively smoking about their general interest to quit, the large majority (approximately 70%) indicated that they were interested in quitting (Supplementary Table S2).

### 3.6. Comparison of Smoking Characteristics between Current Smokers Who Cessated and Those Who Continued to Smoke

**Figure 4:**
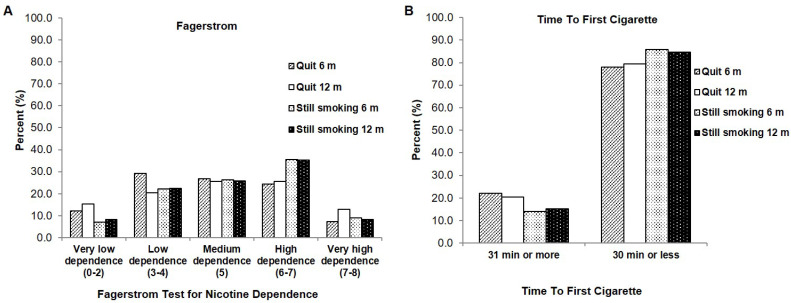
Comparison of smoking variables between those who were smoking and then quit, and those who continued to smoke at 6 months and 12 months (**A**) Fagerstrom Test of Nicotine Dependence (**B**) Time to First Cigarette.

Next, we wanted to examine the smoking variables to identify if there were differences in the smoking characteristics between those who quit smoking after the intensive clinical tobacco intervention, and those who continued to smoke. With regards to the Fagerstrom Test of Nicotine Dependence, those currently smoking and who had continued to smoke in our study had higher dependency scores on the scale, with more frequent incidences of ‘high dependence’ (approximately 35% at both 6- and 12-months) compared to those who had quit and were classified as ‘low-dependence’ (29.3%, 6 months) or ‘medium dependence (26.8%, 6 months) (Figure 4A). Similarly, on the Time To First Cigarette metric, a larger proportion of those who were still smoking (85.9% and 84.7% at 6- and 12-months) had a time to first cigarette of 30 min or less compared to those who were able to quit (78% to 79.5% at 6- and 12-months) (Figure 4B).

### 3.7. Participants Who Had Cessated Prior and Resumed Smoking

There were also instances where those who were cessated at the start of the study resumed smoking again at either the 6- or 12-month follow-up, although these numbers were not high (*n* = 8; 0.03% at 6 months and *n* = 5; 0.02% at 12 months; Supplementary Table S1). When queried about the reasons that they resumed smoking again, the answers were fairly equally attributed to either ‘addiction/habit’ or ‘increased stress’, and they acknowledged that their primary reason to quit again was health (Supplementary Table S3). In addition, there were also some instances where those who were smoking and who had quit after receiving the clinical tobacco intervention at the 6-month interval, had resumed smoking again at the 12-month follow-up (*n* = 7, 0.04%).

## 4. Discussion

### 4.1. Cessation Rate, Cessation Attempts, and Cessation Methods

The purpose of this study was to examine the smoking characteristics of patients with head and neck cancer who had a history of smoking, and also assess the smoking cessation rates in those who were currently smoking after receiving an intensive clinical tobacco intervention that utilized the 3A’s—Ask, Advise, Arrange—and the ‘Opt-Out’ approach prior to receiving their cancer treatment. All patients were contacted at 6- and 12-months post-treatment and asked about their smoking status since the start of the study. The findings demonstrated that at 6 months, 23.7% of those who were smoking had successfully cessated after the intervention, and it had taken between 1–5 attempts to quit smoking before being able to quit completely. Most patients chose to quit ‘cold turkey’ (approximately 50%), although pharmacotherapy (varenicline (Champix)) was used by approximately 35% of the patients in our study.

Our cessation rate (23.7%) is lower in our population of head and neck cancer patients than what has been previously reported [16,17,18,19,20,21]. Tang et al. [16] reported a 36% quit rate after a smoking intervention in head and neck cancer patients 4 months after a cessation intervention, while a cessation rate of 38% was found in general cancer patients [17]. Similarly, higher self-reported abstinence rates (45.8% at 6 months and 43.7% at 9 months [19]; 68% at 6 months [18]) were observed in tobacco treatment programs also offered within a cancer centre [18,19]. With regards to the discrepancy between our study and published findings, demographic factors may explain the differences in cessation/abstinence rates. Specifically, our patient population age is older (median of 67 years) and our Fagerstrom test scores are also higher (29.2% of our patient population scored 6–7, the high dependency bracket, on the intake form). The higher levels of nicotine dependence observed in our patients alone would understandably make cessation a more challenging endeavor.

In terms of cessation attempts, the patients in our study that successfully cessated required between 1–5 attempts before being able to successfully quit. Similar results were reported in a study of former smokers who required between 1–3 attempts to quit [22], and 3.2 attempts were required to quit within 12 months in a head and neck cancer population [23]. Those who were smoking in our study had attempted to quit multiple times prior to being presented the smoking tobacco intervention observed at the baseline timepoint of our study (see [5] and Supplementary Table S3); a finding also shared by others [1].

Approximately half of those who were currently smoking in our study who were able to cessate after receiving the intensive clinical tobacco intervention chose to quit ‘cold turkey’, followed next by pharmacotherapy aids, even though ‘cold turkey’ is a cessation method not endorsed by our intensive intervention program because of high relapse rates. Similarly, Khariwala et al. [24], reported that cold turkey was the first method of choice employed in their study, followed by NRT, and varenicline. Although the option of using these quitting aids was made available to our head and neck cancer patients during the clinical tobacco intervention at our cancer centre, choosing to quit ‘cold turkey’ instead of opting for assistance to quit smoking could be due to factors related to expenses or insurance coverage [22]. Additionally notable is the finding that those who were smoking and who quit self-reported that they used no counselling methods to quit even though all would have received counselling at the cancer clinic from the Intensive Clinical Tobacco Intervention. It is possible that offering the intervention in conjunction with their cancer treatment was not perceived as counselling when questioned about the cessation methods they used to help them quit at follow-up. The proportion of our patients that opted to choose pharmacology (35%) is consistent with others who have also noted that between 26–29% of lung and head and neck cancer patients had chosen pharmacotherapy as a cessation aid [25], while 30% had also used pharmacotherapy in their most recent attempt to quit smoking [23].

### 4.2. Timing of the Clinical Tobacco Intervention and Motivation to Quit

The timing of when clinical tobacco intervention is offered has emerged as a critical factor in successful smoking cessation efforts. One meta-analysis study found that interventions presented peri-operatively compared to cessation interventions offered in the clinic had a pooled OR of 2.31 [26]. Gritz et al. [21] observed that when smoking cessation occurred early (during the first month of their head and neck cancer study) subjects remained cessated; a finding also echoed in the present study. Lower smoking relapse rates in head and neck cancer patients were also found when patients quit prior to receiving surgery, suggesting that the optimal time to present a clinical tobacco intervention is early on in the cancer treatment trajectory, potentially at diagnosis and/or shortly after surgery [27]. Our study utilized the Opt-Out approach [6] where all patients who were smoking were offered a smoking cessation intervention regardless of being enrolled in this study, and the clinical tobacco intervention occurred prior to beginning, or at the beginning of receiving treatment for their head and neck cancer.

Those who were currently smoking in this study indicated that their primary reason for quitting was ‘health’ and may have been motivated to quit at the baseline intake of our study due to a teachable lesson/teachable moment in light of a recent cancer diagnosis, as others have noted [16,28]. This response could be influenced by their diagnosis and treatment for head and neck cancer; however, in a study that also asked both current and former smokers about their reason to quit, most had also cited health concerns [22]. One important finding of our study is that the majority of those smoking expressed interest in quitting (70% at 6 months and 59% at 12 months) despite still continuing to smoke. This finding is consistent with what we reported for the baseline timepoint where 85.8% of this cohort indicated that they were interested in quitting smoking [5]. It is also possible that offering the clinical tobacco intervention and the assistance to quit (counselling and/or pharmacological) could have increased the motivation to quit, a finding also observed by others [4].

### 4.3. Relapse and Longer Time Intervals for Follow-Up

The results of our study also show the challenges surrounding successful smoking cessation, particularly within the first 6 to 12 months of a recent quit attempt. In some instances, patients who were smoking and had cessated by the 6-month follow-up had resumed smoking again at the 12-month follow-up, and some who were cessated at the beginning of the study had also begun smoking again at the 6-month timepoint (*n* = 8). Even though there were a small number of patients that fell into these aforementioned categories, these findings accentuate the need for active follow-ups and monitoring, particularly within the first year of a smoking cessation attempt in patients with head and neck cancer. Studies have reported that the highest relapse rates occur early in a cessation attempt, generally within the first month to 6 months of cessation [27,28,29,30]. The reasons for a potential relapse are multi-faceted. Some patients may not have information on how to prevent a relapse and, additionally, the higher levels of nicotine dependence observed in tobacco-related cancers is also a contributing factor [29,31]. Others have also noted that contributing factors to a smoking relapse could include a lessened confidence in the ability to quit (self-efficacy), lower motivation to quit, withdrawal symptoms, and the particular method used to quit smoking [21,26,27,29,32]. In the patients that accepted the clinical tobacco intervention and had cessated at the 6-month follow-up, our findings show they remained cessated at the 12-month timepoint (with the exception of 7 patients, as noted above). Thus, providing counselling in both the short-term and long-term to circumvent relapse is important [1,32].

### 4.4. Smoking Characteristics

The population of head and neck cancer patients in this study have a long history of smoking and higher levels of nicotine dependence. Those who were smoking and who had cessated in our study reported higher cravings to smoke compared to ex-smokers; similar findings were reported by Gritz et al. [29]. Cigarette cravings were the most common symptom in head and neck cancer patients who had cessated [1,29], followed by restlessness, irritability and anxiety [1]. Additionally, Cooley et al. [25] found that cravings, as well as self-efficacy (belief in the ability to quit), were the variables that predicted abstinence in their study of both lung and head and neck cancer patients. The finding that those who had cessated prior in our study reported few cravings or urges was expected given the fact that the large majority of those patients in our study cohort were not recent quitters and at least 43% of our cohort had been cessated for at least 20 years. For those who were smoking and who were able to cessate, future studies that employ longer follow-up time intervals will have to examine if those cravings and urges experienced by the patients in our study will diminish and become less intense the longer a patient remains cessated.

### 4.5. Study Limitations

There are some limitations observed in the present study. First, the motivation to quit in our patients who were currently smoking was high, and these patients may have been motivated to give answers that would appear better to the interviewer since the questionnaire was not completed independently. Second, the questionnaires employed in the study could be subject to self-report recall error and bias. Further, all interviews and follow-ups were performed by the same individual in our study, which may present some bias. However, we argue that this would also provide an additional level of familiarity for the patients at subsequent follow-up times, and would also provide consistency during the data acquisition portion of the study. Lastly, we do not have a biochemical verification of smoking status, but many studies have found self-reported smoking abstinence to be consistent with biochemical analysis. Cincirpini et al. [19] found that there was high consistency between self-reports of not smoking and expired carbon monoxide levels (between 87–93% on as many as 8877 assessments). A high concordance (93.9%) between self-reported cessation and biochemical verification has also been reported in a meta-analysis study [26], and high concordance rates [21,33] have similarly been reported between self-reported cessation and biochemical confirmation.

### 4.6. Future Directions

One of the future directions of this study would be to extend the clinical tobacco intervention program beyond head and neck cancer patients. With the future implementation of digital medical records systems, referral program will be able to include all smokers regardless of cancer type that attend the Northeast Cancer Centre for their cancer treatment [34,35]. Additional and ongoing concurrent studies within this patient population are also examining the cost barriers and the impact of supplemental funding for nicotine replacement therapy or pharmacological aids, to identify possible barriers to a successful smoking cessation effort.

## Figures and Tables

**Table 1:** Sociodemographic characteristics of head and neck cancer patients (*n* = 503) by smoking status (currently smoking (*n* = 186) and cessated prior (former or ex-smoker (*n* = 317)).

	Overall	Smoking	Cessated
	*n* = 503 (*n*%)	*n* = 186 (*n*%)	*n* = 317 (*n*%)
***Sex***			
Male	389 (77.3)	142 (76.3)	247 (77.9)
Female	114 (22.7)	44 (23.7)	70 (22.1)
***Age***			
Median (Range)	67 (37–96)	64 (38–90)	69 (37–96)
***Marital Status***			
Single	72 (14.3)	42 (22.6)	30 (9.5)
Married or Common-Law	321 (63.8)	96 (51.6)	225 (71.0)
Separated or Divorced	21 (4.2)	13 (7.0)	8 (2.5)
Widow/Widower	63 (12.5)	26 (14.0)	37 (11.7)
Data not available	24 (4.8)	9 (4.8)	15 (4.7)
***ICD Diagnosis***			
Oral Cavity (C01–C06)	186 (37.0)	82 (44.1)	104 (32.8)
Oropharynx (C09–C10)	59 (11.7)	21 (11.3)	38 (12.0)
Larynx (C32)	91 (18.1)	34 (18.3)	57 (18.0)
ICD codes not included above	143 (28.4)	40 (21.5)	103 (32.5)
D00–D44	15 (3.0)	5 (2.7)	10 (3.2)
***Stage***			
T1	120 (23.9)	43 (23.1)	77 (24.3)
T2	154 (30.6)	62 (33.3)	92 (29.0)
T3	80 (15.9)	30 (16.1)	50 (15.8)
T4	75 (14.9)	33 (17.7)	42 (13.2)
TX	9 (1.8)	5 (2.7)	n/a
*Missing*	62 (12.3)	12 (6.5)	50 (15.8)
***Physical Comorbidities***			
None	171 (24.2)	78 (31.8)	93 (20.1)
Diabetes	75 (10.6)	17 (6.9)	58 (12.5)
Hypertension	196 (27.7)	55 (22.4)	141 (30.5)
Heart problems/heart failure	82 (11.6)	25 (10.2)	57 (12.3)
Lung/COPD/Asthma	52 (7.3)	23 (9.4)	29 (6.3)
Stroke/circulatory	19 (2.7)	7 (2.9)	12 (2.6)
Other cancers	105 (14.8)	36 (14.7)	69 (14.9)
Data not available	8 (1.1)	n/a	n/a
	*n* = 501; 708 responses	*n* = 186; 245 responses	*n* = 315; 463 responses
***Psychiatric Comorbidities***			
None	462 (91.8)	166 (89.2)	296 (93.4)
Depression	19 (3.8)	11 (5.9)	8 (2.5)
Anxiety	9 (1.8)	n/a	7 (2.2)
Depression & Anxiety	8 (1.6)	5 (2.7)	n/a
***Alcohol Consumption***			
Yes	300 (59.6)	121 (65.1)	179 (56.5)
No	137 (27.2)	47 (25.3)	90 (28.4)
Data not available	64 (12.7)	18 (9.7)	46 (14.5)
***Alcohol Dependency***			
Yes	62 (12.3)	42 (22.6)	20 (6.3)
No	374 (74.4)	125 (67.2)	249 (78.5)
Data not available	65 (12.9)	19 (10.2)	46 (14.5)
***Drug Use***			
Yes	6 (1.2)	n/a	n/a
No	487 (96.8)	178 (95.7)	309 (97.5)
Data not available	7 (1.4)	n/a	n/a

Note: Cells with *n* < 5 have been suppressed, indicated by n/a.

**Table 2:** Smoking characteristics of head and neck cancer patients (*n* = 503) who are current smokers (*n* = 186) or had cessated (*n* = 317).

	Overall	Smoking	Cessated	OR ^1^	Adj OR ^2^
	*n* = 503 (*n*%)	*n* = 186 (*n*%)	*n* = 317 (*n*%)	(95% CI)	(95% CI)
***Age started to smoke***					
Median (Range)	16 (4–60)	15 (4–59)	16 (4–60)		
***Number of years cessated* (*Ex-Smokers only*)**					
0–1 years			8 (2.5)		
2–5 years			70 (22.1)		
6–10 years			45 (14.2)		
11–20 years			56 (17.7)		
21–30 years			45 (14.2)		
31–40 years			55 (17.4)		
41+ years			36 (11.4)		
*Missing*			2 (0.6)		
***Duration Smoked (years)***					
1–30	193 (38.4)	28 (15.1)	165 (52.1)	*1.0 ref*	*1.0 ref*
31–43	146 (29.0)	59 (31.7)	87 (27.4)	3.97 (2.38–6.72)	4.22 (2.30–7.74)
44–75	163 (32.4)	98 (52.7)	65 (20.5)	8.89 (5.34–14.78)	19.59 (9.94–38.59)
***Number of Cigarettes Smoked per Day***					
10 or less	83 (16.5)	15 (8.1)	68 (21.5)	*1.0 ref*	*1.0 ref*
11–20	199 (39.6)	83 (44.6)	116 (36.6)	3.24 (1.73–6.07)	3.44 (1.71–6.92)
21–30	147 (29.2)	65 (34.9)	82 (25.9)	3.59 (1.88–6.86)	3.70 (1.79–7.65)
31 or more	71 (14.1)	23 (12.4)	48 (15.1)	2.17 (1.03–4.59)	2.18 (0.93–5.12)
***Smoking Pack Years***					
25 or less	181(36.0)	35 (18.8)	146 (46.1)	*1.0 ref*	*1.0 ref*
26–50	183 (36.4)	84 (45.2)	99 (31.2)	3.54 (2.21–5.66)	4.25 (2.47–7.29)
51 or higher	136 (27.0)	67 (36.0)	69 (21.8)	4.05 (2.46–6.67)	6.16 (3.34–11.36)
***Fagerstrom Test for Nicotine Dependence***					
Very Low Dependence (0–2)	100 (19.9)	14 (7.5)	86 (27.1)	*1.0 ref*	*1.0 ref*
Low Dependence (3–4)	126 (25.0)	47 (25.3)	79 (24.9)	3.66 (1.87–7.15)	3.73 (1.82–7.65)
Medium Dependence (5)	87 (17.3)	44 (23.7)	43 (13.6)	6.29 (3.11–12.71)	5.45 (2.57–11.52)
High Dependence (6–7)	147 (29.2)	63 (33.9)	84 (26.5)	4.61 (2.40–8.85)	3.91 (1.92–7.95)
Very High Dependence (8–10)	38 (7.6)	18 (9.7)	20 (6.3)	5.53 (2.36–12.95)	3.98 (1.55–10.25)
*Missing*	5 (1.0)		5 (1.6)		
***Heaviness of Smoking Index***					
Very Low Dependence	139 (27.6)	27 (14.5)	112 (35.3)	*1.0 ref*	*1.0 ref*
Low–Moderate Dependence	116 (23.1)	56 (30.1)	60 (18.9)	3.87 (2.22–6.75)	3.52 (1.92–6.44)
Moderate Dependence	114 (22.7)	47 (25.3)	67 (12.1)	2.91 (1.66–5.10)	2.57 (1.38–4.77)
Very High Dependence	132 (26.2)	56 (30.1)	76 (24.0)	3.06 (1.77–5.27)	2.59 (1.42–4.75)
***Time to First Cigarette***					
31 or more min	145 (28.8)	29 (15.6)	116 (36.6)	*1.0 ref*	*1.0 ref*
30 min or less	356 (70.8)	157 (84.4)	199 (62.8)	3.16 (2.00–4.99)	2.67 (1.62–4.40)

^1^ Crude OR and 95% CIs. ^2^ Age, gender, marital status, psychiatric comorbidities, alcohol dependency, alcohol consumption, and drug use adjusted OR and 95% CI. Note: Cells with *n* < 5 have been suppressed.

## Data Availability

Data sharing not applicable due to privacy and ethical restrictions.

## Supplementary Materials

The following are available online at https://www.mdpi.com/article/10.3390/curroncol29030130/s1, Figure S1: Consort Flow chart of the head and neck cancer participants. (A) From baseline to 6-month follow-up. (B) from baseline to 12-month follow-up. Table S1: Questionnaire presented to all ever-smokers at the 6 and 12 month follow-up timepoints. Table S2: Interest and motivations to quit smoking in those currently smoking who continued smoking after receiving a tobacco cessation intervention at 6 and 12 months. Table S3: Smoking data from former or ex-smokers who were cessated at the beginning of the study who resumed smoking at the 6 month and 12 month follow-up.
