# Supplementary material for: Rates of Smoking Cessation at 6 and 12 Months after a Clinical Tobacco Smoking Cessation Intervention in Head and Neck Cancer Patients in Northern Ontario, Canada
Source: Curr Oncol. 2022 Mar 2;29(3):1544–58. doi: 10.3390/curroncol29030130 (PMC8947430; doi:10.3390/curroncol29030130)
Supplement: Supplementary file 1 [file curroncol-29-00130-s001.zip › curroncol-1495078-supplementary.pdf]

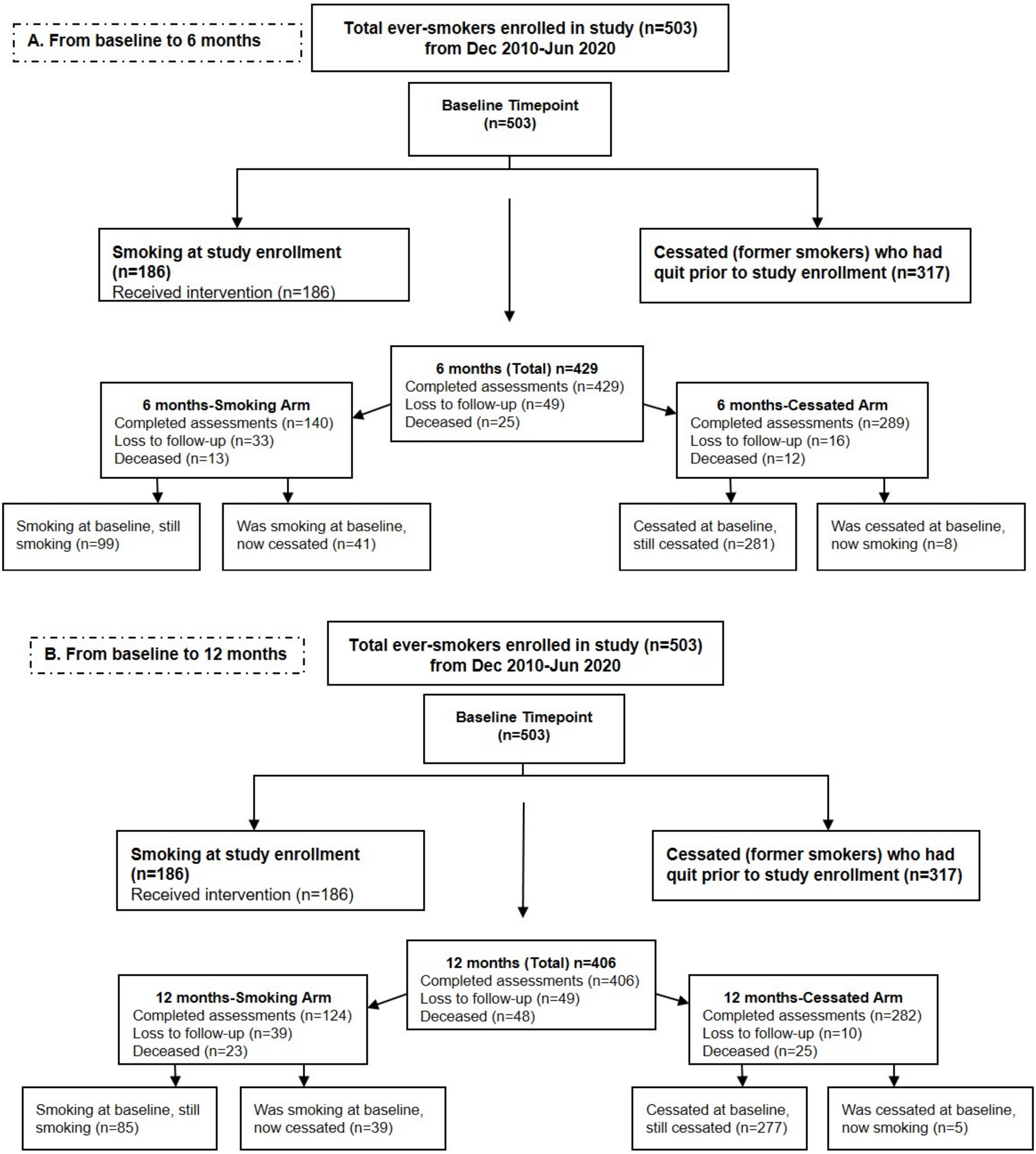

Figure S1. Consort Flow chart of the head and neck cancer participants (A) From baseline to 6 month follow-up (B) from baseline to 12 month follow-up.

Table S1. Questionnaire presented to all ever-smokers at the 6 and 12 month follow-up timepoints.

From our initial Tobacco Use Questionnaire, you indicated at that present time you:

€ **Were currently smoking cigarettes**		€ **Had completely quit smoking**	
**Are you still currently smoking cigarettes?**		**Are you still completely cessated?**	
€ **No**	€ **Yes**	€ **No**	€ **Yes**
On what date did you quit smoking?<br>______(Date quit)	How many cigarettes are you currently smoking a day<br>______(Per/Day)	On what date did you start smoking again?<br>______(Date re-started)	Do you have any cravings or urges to have a cigarette on a regular basis?<br>€ No<br>€ Yes
How many attempts did you make before you were able to successfully quit?<br>______(Attempts)	Have you attempted to quit since you completed the initial questionnaire<br>€ No<br>€ Yes	How many cigarettes per day are you currently smoking?<br>______(Per/Day)	How would you rate your urge to have a cigarette?<br>€ Extreme urge<br>€ Moderate urge<br>€ Mild Urge<br>€ None
What cessation method(s) did you use in your attempts to quit smoking?<br>€ NRT<br>€ Champix<br>€ Zyban<br>€ Counselling<br>€ None (Cold Turkey)<br>€ Other______	How many attempts did you make?<br>_____(Attempts)<br>How long did you last before having a cigarette?<br>____(Days)<br>____(Months)<br>____(Years)	What factors do you believe lead you to begin smoking again?<br>€ Addiction/habit<br>€ Increased stress<br>€ Being around other smokers<br>€ Other________<br>_____________	**Thank you,**<br>**Follow-up assessment complete**
Do you have any cravings or urges to have a cigarette on a regular basis?<br>€ No<br>€ Yes	Are you **presently** interested in quitting smoking?<br>€ No<br>€ Yes	Are you **presently** interested in quitting smoking?<br>€ No<br>€ Yes	

How would you rate your urge to have a cigarette?<br>€ Extreme urge<br>€ Moderate urge<br>€ Mild urge<br>€ None	What are your **motivations** for quitting smoking?<br>€ Health<br>€ Pregnancy or baby<br>€ Cost of cigarettes<br>€ Less stress in life<br>€ Smoking is less acceptable nowadays<br>€ Other_____	What are your **motivations** for quitting smoking?<br>€ Health<br>€ Pregnancy or baby<br>€ Cost of cigarettes<br>€ Less stress in life<br>€ Smoking is less acceptable nowadays<br>€ Other_____	
**Thank you,**<br>**Follow-up assessment complete**	Are you seriously considering quitting smoking within the next **30 days?**<br>€ No<br>€ Yes	Are you seriously considering quitting smoking within the next **30 days?**<br>€ No<br>€ Yes<br>€	
	Are you seriously considering quitting smoking within the next **6 months?**<br>€ No<br>€ Yes	Are you seriously considering quitting smoking within the next **6 months?**<br>€ No<br>€ Yes	
	What cessation strategies and/or products do you believe will help you successfully quit?<br>€ Motivational counselling<br>€ Motivational support from family or loved ones<br>€ NRT<br>€ Prescription medication<br>€ None (Cold Turkey)<br>€ Other	What cessation strategies and/or products do you believe will help you successfully quit?<br>€ Motivational counselling<br>€ Motivational support from family or loved ones<br>€ NRT<br>€ Prescription medication<br>€ None (Cold Turkey)<br>€ Other	
	**Thank you,**<br>**Follow-up assessment complete**	**Thank you,**<br>**Follow-up assessment complete**	

Table S2. Interest and motivations to quit smoking in those currently smoking who continued smoking after receiving a tobacco cessation intervention at 6 and 12 months

Baseline	*6 months n (%)*	*12 months n (%)*
**Smoking (n=186)**	**Smoking (n=99)**	**Smoking (n=85)**
Present interest in quitting smoking		
No	28 (28.3)	26 (30.6)
Yes	70 (70.7)	59 (69.4)
Serious consideration to quit within the next 30 days		
No	44 (44.4)	42 (49.4)
Yes	51 (51.5)	39 (45.9)
Serious consideration to quit within the next 6 months		
No	21 (21.2)	20 (23.5)
Yes	73 (73.7)	60 (70.6)
Missing	5 (5.1)	5 (5.9)
Motivations for quitting smoking		
Health	74 (82.2)	64 (82.1)
Pregnancy or baby	n/a	n/a
Cost of cigarettes	6 (6.7)	n/a
Less stress in life	n/a	n/a
Smoking is less acceptable today	n/a	n/a
Other	n/a	n/a
No motivation to quit	5 (5.6)	11 (14.1)
	n=84; 90 responses	n=76; 78 responses
Cessation strategies or products that will enable successful cessation		
Motivational counselling	n/a	n/a
Motivational support	n/a	n/a
Nicotine replacement therapy	13 (12.1)	17 (17.5)
Prescription medication	35 (32.7)	35 (36.1)
None (“Cold Turkey”)	43 (40.2)	33 (34.0)
Other	12 (11.2)	10 (10.3)
	n=91; 107 responses	n=81; 97 responses

Note: Cells with n<5 have been suppressed, indicated by n/a

Table S3. Smoking data from former or ex-smokers who were cessated at the beginning of the study who resumed smoking at the 6 month and 12 month follow-up.

Baseline	*6 months n (%)*	*12 months n (%)*
**Cessated (n=317)**	**Smoking (n=8)**	**Smoking (n=5)**
Number of cigarettes currently smoked per day		

0-10 cigarettes	7 (87.5)	5 (100.0)
11 and higher	n/a	n/a
Factors that lead to resumption of smoking		
Addiction/habit	7 (58.3)	3 (60.0)
Increased stress	5 (41.7)	2 (40.0)
Being around other smokers	n/a	n/a
Other	n/a	n/a
	n=7; 12 responses	n=5; 5 responses
Present interest in quitting smoking		
No	2 (25.0)	2 (40.0)
Yes	6 (75.0)	3 (60.0)
Serious consideration to quit within the next 30 days		
No	2 (25.0)	2 (40.0)
Yes	6 (75.0)	3 (60.0)
Serious consideration to quit within the next 6 months		
No	n/a	n/a
Yes	7 (87.5)	4 (80.0)
Motivations for quitting smoking		
Health	6 (75.0)	4 (100.0)
Pregnancy or baby	n/a	n/a
Cost of cigarettes	n/a	n/a
Less stress in life	n/a	n/a
Smoking is less acceptable today	n/a	n/a
Other	n/a	n/a
No motivation to quit	2 (25.0)	n/a
Cessation strategies or products that will enable successful cessation		
Motivational counselling	n/a	n/a
Motivational support	n/a	n/a
Nicotine replacement therapy	2 (20.0)	n/a
Prescription medication	5 (50.0)	2 (40.0)
None (“Cold Turkey”)	2 (20.0)	n/a
Other	n/a	2 (40.0)
	n=8; 10 responses	n=5; 5 responses
